# Novel Eco-friendly, One-Pot Method for the Synthesis of Kynurenic Acid Ethyl Esters

**DOI:** 10.1021/acsomega.3c01170

**Published:** 2023-05-11

**Authors:** Péter Simon, Bálint Lőrinczi, Anasztázia Hetényi, István Szatmári

**Affiliations:** † Institute of Pharmaceutical Chemistry, University of Szeged, Eötvös u. 6, H-6720 Szeged, Hungary; ‡ Department of Medical Chemistry, University of Szeged, Dóm tér 8, H-6720 Szeged, Hungary; § Stereochemistry Research Group, Eötvös Loránd Research Network, University of Szeged, Eötvös u. 6, H-6720 Szeged, Hungary

## Abstract

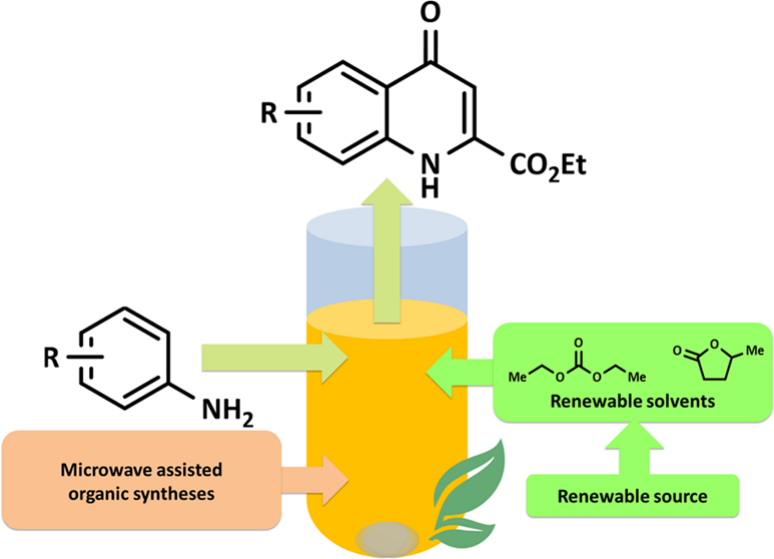

The synthesis of kynurenic acid derivatives with potential biological effect was investigated and optimized for one-batch, two-step microwave-assisted reactions. Utilizing both chemically and biologically representative non-, methyl-, methoxy-, and chlorosubstituted aniline derivatives, in catalyst-free conditions, syntheses of seven kynurenic acid derivatives were achieved in a time frame of 2–3.5 h. In place of halogenated reaction media, tuneable green solvents were introduced for each analogue. The potential of green solvent mixtures to replace traditional solvents and to alter the regioisomeric ratio regarding the Conrad–Limpach method was highlighted. The advantages of the fast, eco-friendly, inexpensive analytic technique of TLC densitometry were emphasized for reaction monitoring and conversion determination in comparison to quantitative NMR. Moreover, the developed 2–3.5 h syntheses were scaled-up to achieve gram-scale products of KYNA derivatives, without altering the reaction time in the halogenated solvent DCB and more importantly in its green substitutes.

## 1 Introduction

Since the 12 principles of green chemistry were established in 1989 by Anastas and Warner,^1^ several measures have been taken to develop environmentally benign and safer procedures in the field of preparative organic chemistry and analytics. The most effective action to decrease the environmental impact of chemical synthesis is either the replacement of traditional solvents with neat systems or the use of green substitutes.^1−10^

Biobased solvents have been in the focus of the development of green processes, and extensive literature has introduced dialkyl carbonates, such as diethyl carbonate (DEC) and γ-valerolactone (GVL), as sustainable liquids and/or solvents. In the field of biofuel production also, using these compounds as solvents would be a good alternative.^9,11−20^

Compounds of great biological relevance are often synthesized via non-eco-friendly methods. One such molecule is kynurenic acid (KYNA), an endogenous heterocyclic compound derived from L-tryptophane. A deviation from its physiological level contributes to neurological disorders such as Alzheimer’s, Parkinson’s, and Huntington’s diseases, epilepsy, and migraine.^21−26^ Syntheses of numerous KYNA analogues, such as methyl-, halogeno- and methoxy-substituted compounds, have been described in the literature. Most of these approaches utilize the Conrad–Limpach (CL) method or its modifications, in which non-eco-friendly and difficult-to-handle reagents or solvents are used, such as diphenyl ether or polyphosphoric acid.^27−39^

In recent literature, modifications of the CL method have been introduced regarding the heat source, solvent selection, and additional work-up processes.^40,41^ Herein, a novel, one-batch two-step MW-assisted method, using only a single solvent system for the total synthesis of KYNA and its derivatives, is proposed (Table 1).

**Table 1:**
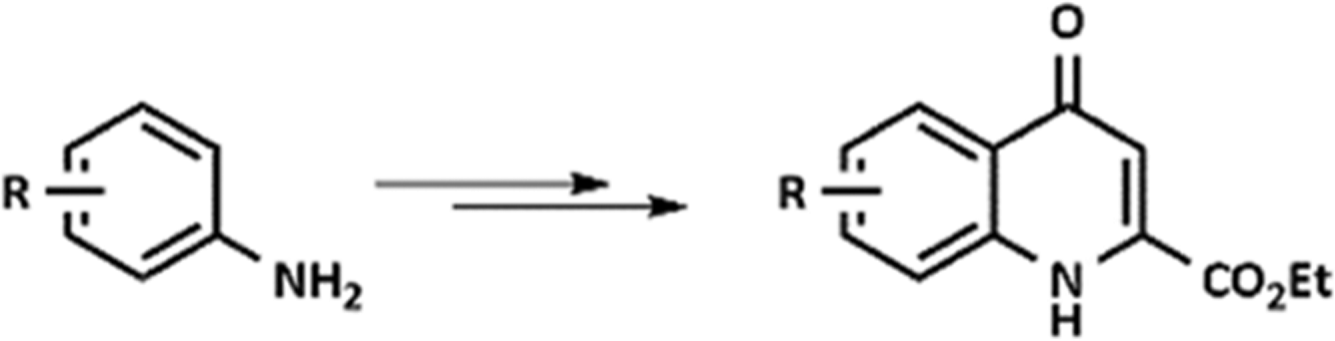
Broadening the Scope of CL Synthesis of Kynurenic Acid Derivatives via Green Chemical Insights

previous work	
R:	conditions or catalysts
H, 6-Me	heat,^32^ PPA^34^
6-MeO	heat^,^^30^^MW^,^41^ PPA^34^
5-MeO	catalyst^35^^a^
7-MeO	PPA,^34^ catalyst^35^*
5-Cl, 7-Cl	heat^36,38^
5-OH, 6-OH, 7-OH	heat, MW, *p*TSOH^40^

this work	
H, 6-Me, 5-MeO, 6-MeO, 7-MeO, 5-Cl, 7-Cl	MW assistance
	tuneable green solvent systems
	modification of regioselectivity

a In the case of the patented literature ref35., limited information was available about the catalyst. All heating methods were conducted in either diphenyl ether^30,32,41^ or 1,2-dichlorobenzene^36,40^

## 2 Results and Discussion

### 2.1 One-Pot, Two-Step Syntheses of KYNA and its Derivatives Using Microwave-Assisted Heating

**Scheme 1:**
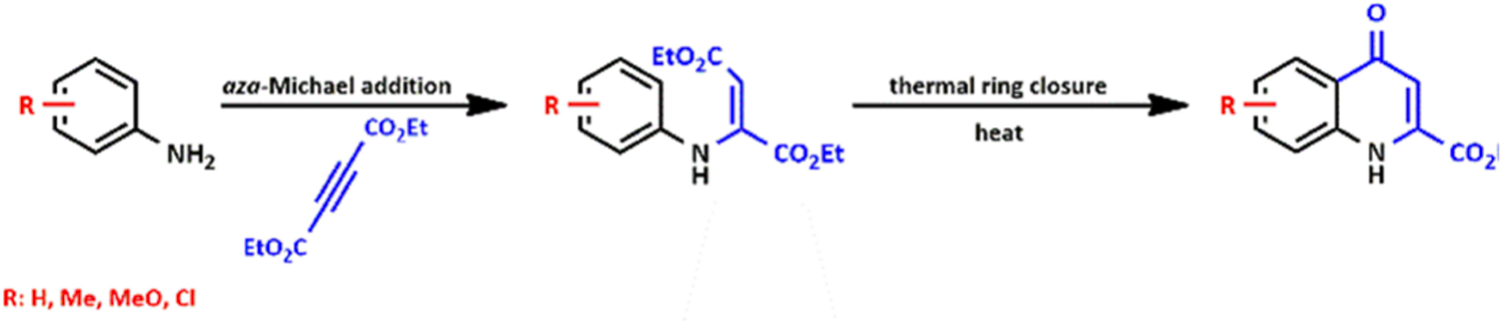
Synthesis of Substituted Kynurenic Acid Ethyl Esters Using the CL Method

The CL method itself consists of two main steps: (i) in the reaction between an aniline derivative and diethyl acetylenedicarboxylate (DEAD), an enamine is formed through an *aza*-Michael addition (*a*MA); (ii) next, the final product is synthesized via thermal ring closure (TRC) through the elimination of an ethanol molecule (Scheme 1).

To limit solvent consumption, neither chromatographic cleaning techniques nor distillation were used between the steps.

Advantages of MW-assisted heating techniques have been reported in the scientific literature, regarding preparative methods or digestion for analysis. Being a closed system, temperatures above boiling points of either solvents or reagents can be achieved, minimizing the environmental impact of the highly irritative starting materials or enamines. Moreover, specific microwave effects take place in MW-conducted reactions. Such (thermal) effects occur due to the overheating effect of solvents, selective heating of reagents of catalysts in the reaction media, formation of hotspots (by direct coupling of reagents), and the bulk-heating model (lack of “wall effect”), which leads to a homologous temperature profile in the reaction chamber. There have been recent debates over such athermal effects of electromagnetic irradiation on dipolar molecules, which can alter the pre-exponential factor or the activation energy of the reaction and not only the temperature. It is important to note that in the case of microwave-assisted syntheses, reaction conditions can be precisely set and handled.^42−45^

#### 2.1.1 Syntheses of C-6-Substituted KYNA Analogues

**Scheme 2:**
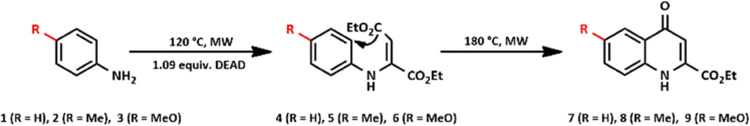
CL Synthesis of C-6-Substituted KYNA Ethyl Esters

Starting from aniline derivatives **1–3**, through the formation of enamines **4–6**, KYNA analogues **7–9** were synthesized (Scheme 2).

**Figure 1:**
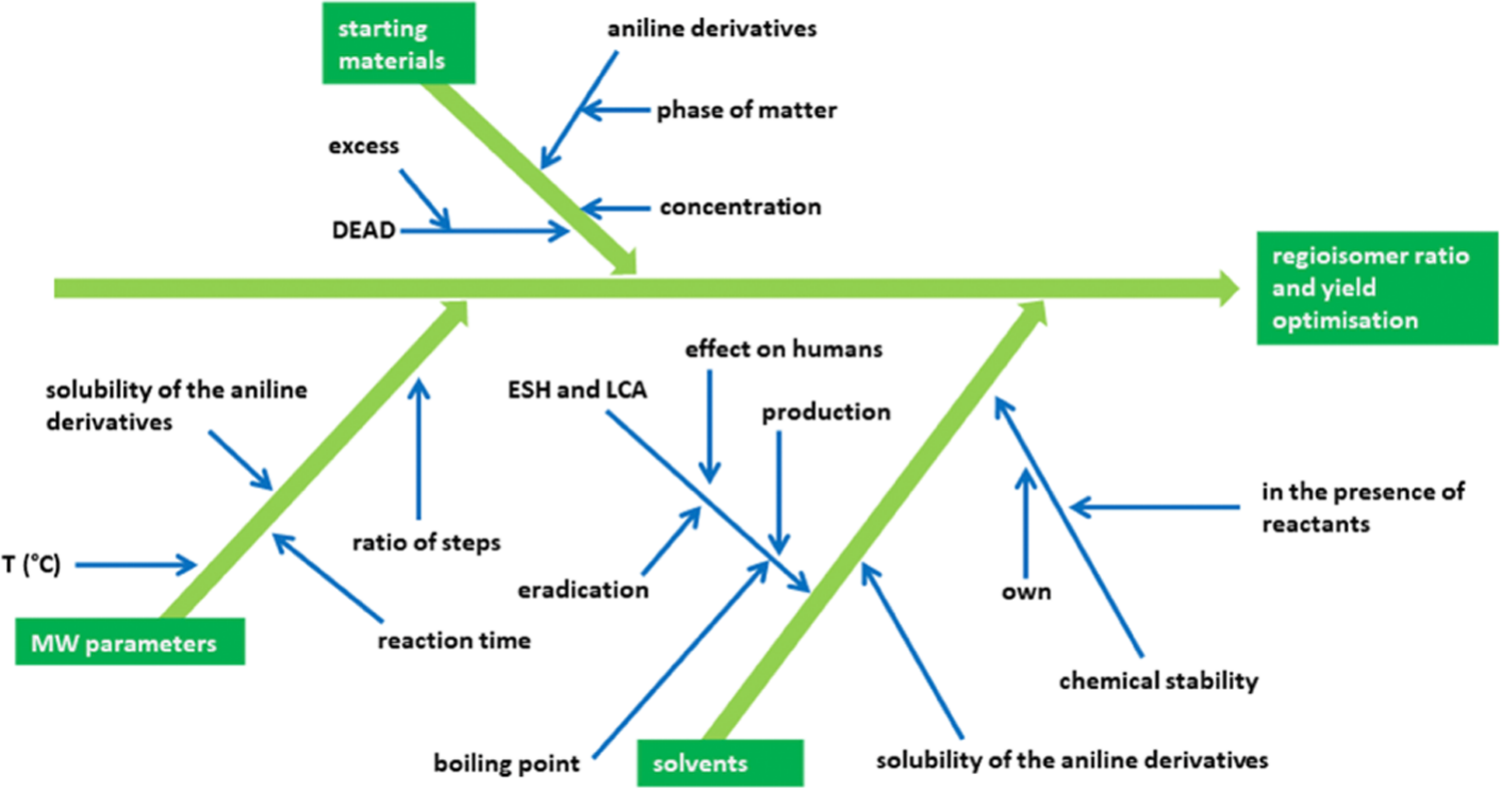
Factors taken into consideration while designing the MW-assisted reactions illustrated through an Ishikawa diagram.

While designing the reaction methods suitable for the one-pot microwave-assisted synthesis, several factors were taken into consideration regarding the starting materials, including MW parameters and solvents (Figure 1).

Reaction conditions of each step were precisely set as follows:

Volume: In each case, a 5.0 mL reaction volume was investigated.

Concentration: In the case of anilines as starting materials, a concentration of 0.5 M of the selected solvents was found to be optimal. At higher concentrations (*e.g.*, 1.5–5 M), maleimide-type side products formed, and the conversion decreased.^39^

Excess: The excess of diethyl acetylenedicarboxylate (DEAD) was determined to be 1.09 equiv, as undesirable side products were occasionally formed above 1.1 equiv.

Solubility: Regarding the starting materials (in the case of solid aniline derivatives), the solubility was taken into consideration, as undissolved fractions of anilines are prone to degradation.

Reaction temperature:

(i) In test reactions utilizing the 1,2-dichlorobenzene (DCB) solvent, 120 °C was found to be the optimal temperature for the aMA reaction step. This was presumably observed due to the slower enamine formation in this media, compared with methods using ethanol. It is important to note that the boiling point of DEAD is 107–110 °C; therefore, a closed reaction system was needed.
(ii) The TRC step of the syntheses was conducted at 180 °C. Temperatures above 180 °C did not lead to significantly higher yields; however, in some cases, side products were formed. It is also important to mention that in order to avoid the formation of maleimides (usually at about 150 °C), heating up from 120 °C to the next phase should be as fast as possible.

Reaction time: For each synthesis, individual reaction times were determined in order to maximize conversion (Table 2).

**Table 2:** Different Reaction Times of Both Steps (i) and (ii) Used in the Synthesis of KYNA Derivatives

	reaction time (min)	
product	aza-Michael addition	thermal ring closure
7	60	60
8	90	120
9	60	60
14a–b	90	60
15a–b	120	60

Green solvent systems: Reaction conditions finally being set, our further experiments focused on the exchange of 1,2-dichlorobenzene (DCB) with green solvents, without altering reaction times. From two solvents (GVL and DEC), selected based on their physicochemical traits [boiling point, stability, environment–safety–health (ESH) data, and life cycle assessment (LCA)], four green solvent systems were prepared. Ultimately, test reactions were conducted in DCB, GVL, DEC, and two mixtures of the latter [1:2 (V27) and 2:1 (V60), given in *n*_GVL_/*n*_DEC_ molar ratios].

#### 2.1.2 Syntheses of C-5- and C-7-Substituted KYNA Analogues

**Scheme 3:**
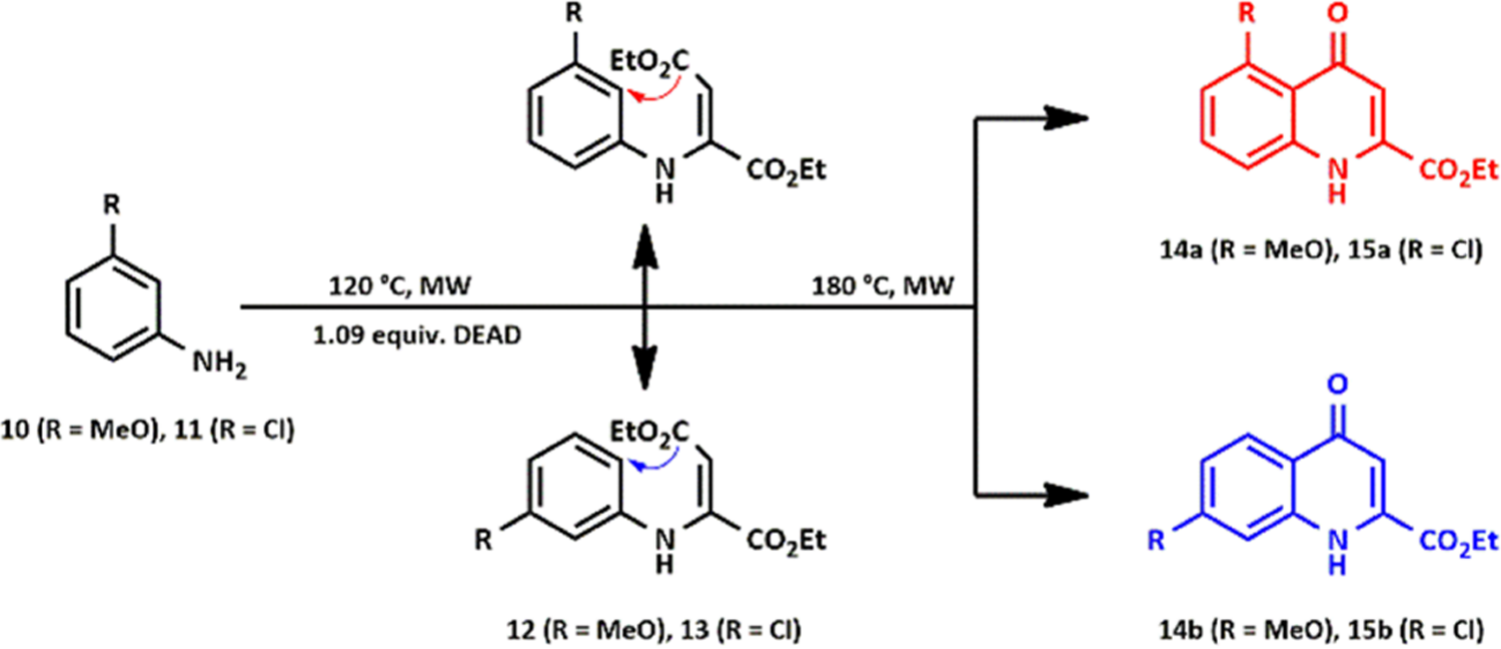
CL Syntheses of C-5- and C-7-Substituted KYNA Ethyl Esters

Further experiments focused on KYNA ethyl esters formed as regioisomer pairs (Scheme 3).

Reaction conditions were set on the basis of the same conclusions as mentioned before, *i.e.*, 0.5 M concentration of the aniline derivative, 5.0 mL reaction volume, 1.09 equiv of DEAD, 120 °C temperature for step (i), and 180 °C for step (ii). Reaction times are summarized in Table 2.

Compound pairs **14a–b** and **15a–b** having been synthesized in DCB, our next aim was to alter the reaction conditions in order to affect the regioisomeric ratios. First, temperatures at which the TRCs were conducted were changed. At 160 °C, no or only trace amounts of products were formed, and above 180 °C, set previously (investigated at every 20 °C temperature increase to 220 °C), no significant change in the regioselectivity was detected, but side products formed.

Results of the experiments (Tables 3 and 4) led to the deduction that regarding the synthesis of unsubstituted, 6-Me-, 5-, 6-substituted, and 7-MeO-substituted, and 5- and 7-Cl-substituted KYNA ethyl esters via the CL method, a green solvent system of GVL and DEC can be prepared for each test reaction in which the synthesis can be conducted with nearly the same (or higher) conversion as that with the use of DCB.

Furthermore, a solvent effect on the regioisomeric ratios was observed in the four green solvent systems (Table 4).

### 2.2 Determination of Conversion

In order to quantify the exact mass of the dissolved products, several methods were taken into consideration. Because of the complexity of the crude product, the HPLC method was suitable but seemed to be problematic due to the costly column. GC methods would not be applicable as the products and matrices are of significantly low volatility.

Therefore, two methods, verifying one another, were considered to be adequate, because of the difference in their physicochemical properties.

#### 2.2.1 Quantitative NMR

**Figure 2:**
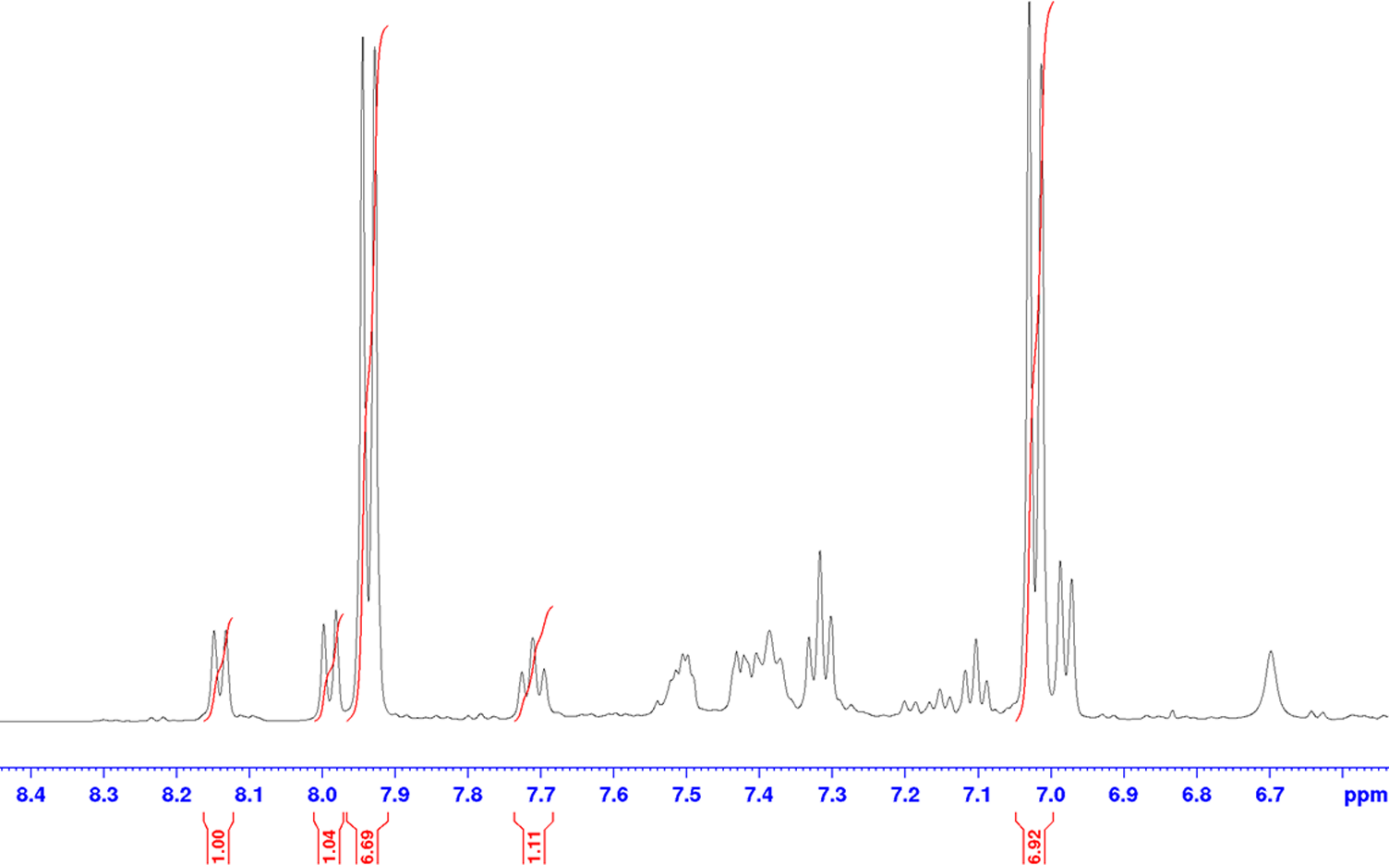
Quantitative NMR spectrum of the crude reaction mixture of the synthesis of compound **7** in GVL using *p-*methoxybenzoic acid as the internal standard (aromatic region).

Quantitative NMR was first chosen to quantify the dissolved products. It is a low-cost, solvent-sparing, rapid method to determine unisolated amounts of products in the reaction media itself. The method relies on the principle of rationalizing peaks: one (or more) of the analyte to one (or more) of the reference compound (internal standard). Beneficial traits of the adequate internal standard are the easily recognizable peaks in the ^1^H NMR spectra, chemical stability in the reaction media, and easy handling.^46−48^ These requirements led to the selection of *p*-methoxybenzoic acid (anisic acid, PAA) purchased from Merck as the internal standard. However, due to the complex matrices and, in some cases, low conversions and/or overlapping peaks, the method was found to be not universal (Figure 2). At high temperatures, the formation of substituted *N*-(4-hydroxy)valeroyl aniline or *N*-ethoxycarbonyl aniline byproducts can be hypothesized, although a thorough analysis of the crude NMR spectra proved that these compounds were below the limit of detection.

#### 2.2.2 TLC Densitometric Measurements

TLC densitometry was found to be a fast, universal, low-cost, and eco-friendly method for determining dissolved products with a limit of quantification of nanograms. Using a single digital camera or a common smartphone with the calibration series, the dissolved quantity of the desired compound can be easily defined with a good signal/noise ratio.

**Figure 3:**
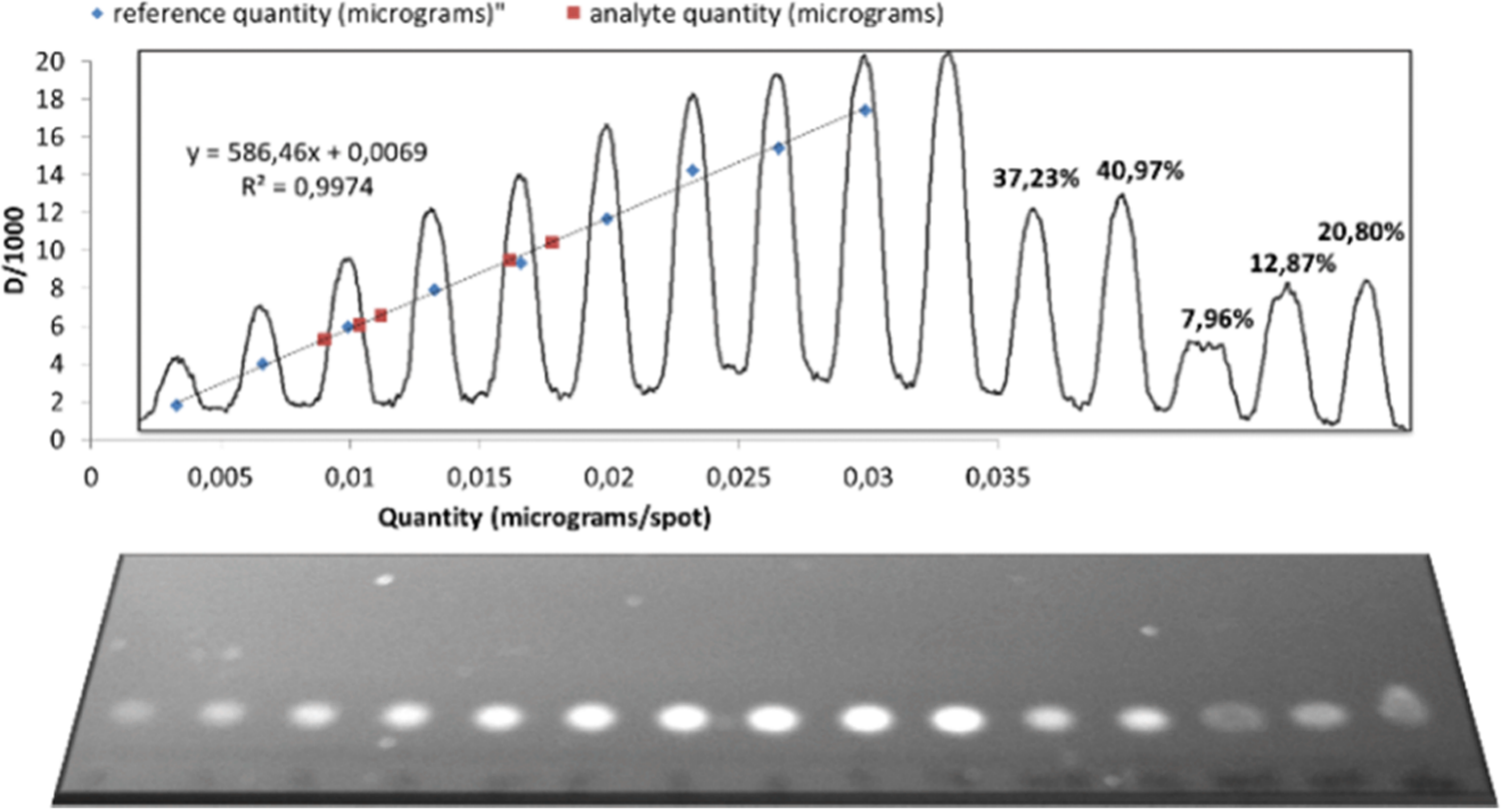
TLC densitometric analysis of compound **7**: record of the plate, densitogram, regression curve on a graph (quantity of desired compound [μg] → optical density divided by 1000), and calculated dissolved products given as conversion × selectivity [%].

The method itself was taken into consideration, because of the great difference in retention factors of the products, side products, and remaining reactants. Furthermore, these compounds have characteristic UV activities. TLC densitometry has been introduced in the scientific literature due to its advantages.^49−52^ The method itself is mostly utilized in the field of pharmacognosy, quantifying bioactive components of drugs and food materials such as L-theanine, histamine, cadaverine, spermidine, tyramine, putrescine, rosmarinic acid, and flavonoids.^49−54^ The authors also introduced new aspects of TLC densitometry; specifically, the use of a densitometer was changed to the use of digital cameras or even newer smartphones with cameras.^55^ The principle of the method relies on the simultaneous elution, derivatization (if needed), and recording of the reference matter (as a calibration series) and the analyte sample on the very same TLC plate. These conditions enable the use of a calibration curve and verify the results calculated with a linear regression equation (Figure 3). It is crucial to find the linear range of the function (quantity of the desired compound [μg] → optical density divided by 1000) as calculations via nonlinear regressions would be disadvantageous.

In summary, TLC densitometry was found to be the universal method to quantify dissolved products, although qNMR, in the case of each test reaction, was able to validate the measurements. The results of compounds **7–9** are summarized in Table 3, while those of **14a–b** and **15a–b** are presented in Table 4.

**Table 3:** Molar Percentages of Dissolved Products Compared to the Corresponding Starting Aniline Derivative [%] in the Synthesis of Compounds 7–9 in DCB, GVL, DEC, V27, and V60 Solvents, Measured by Densitometry and Quantitative NMR of the Crude Products

product	solvent system	dissolved product [%] (densitometry, DM)	dissolved product [%] (qNMR)
7	DCB	33.75	31.50
	GVL	38.34	39.45
	DEC	5.02	4.74
	V27	12.25	12.94
	V60	20.56	23.95
8	DCB	72.28	72.99
	GVL	43.34	38.25
	DEC	12.08	12.28
	V27	56.01	52.66
	V60	73.07	72.16
9	DCB	34.82	48.35^a^
	GVL	9.41	9.88
	DEC	10.61	5.31^a^
	V27	38.09	40.76
	V60	10.85	14.70^a^

a Outliers due to overlapping peaks in the ^1^H NMR spectra of crude products.

The conversions achievable in DCB could be reached or surpassed in the case of the synthesis of each compound. In the case of compounds **7**, **8**, and **9**, GVL, V60, and V27 were, respectively, the optimal green solvents to replace the traditional solvent, while achieving higher conversions.

**Figure 4:**
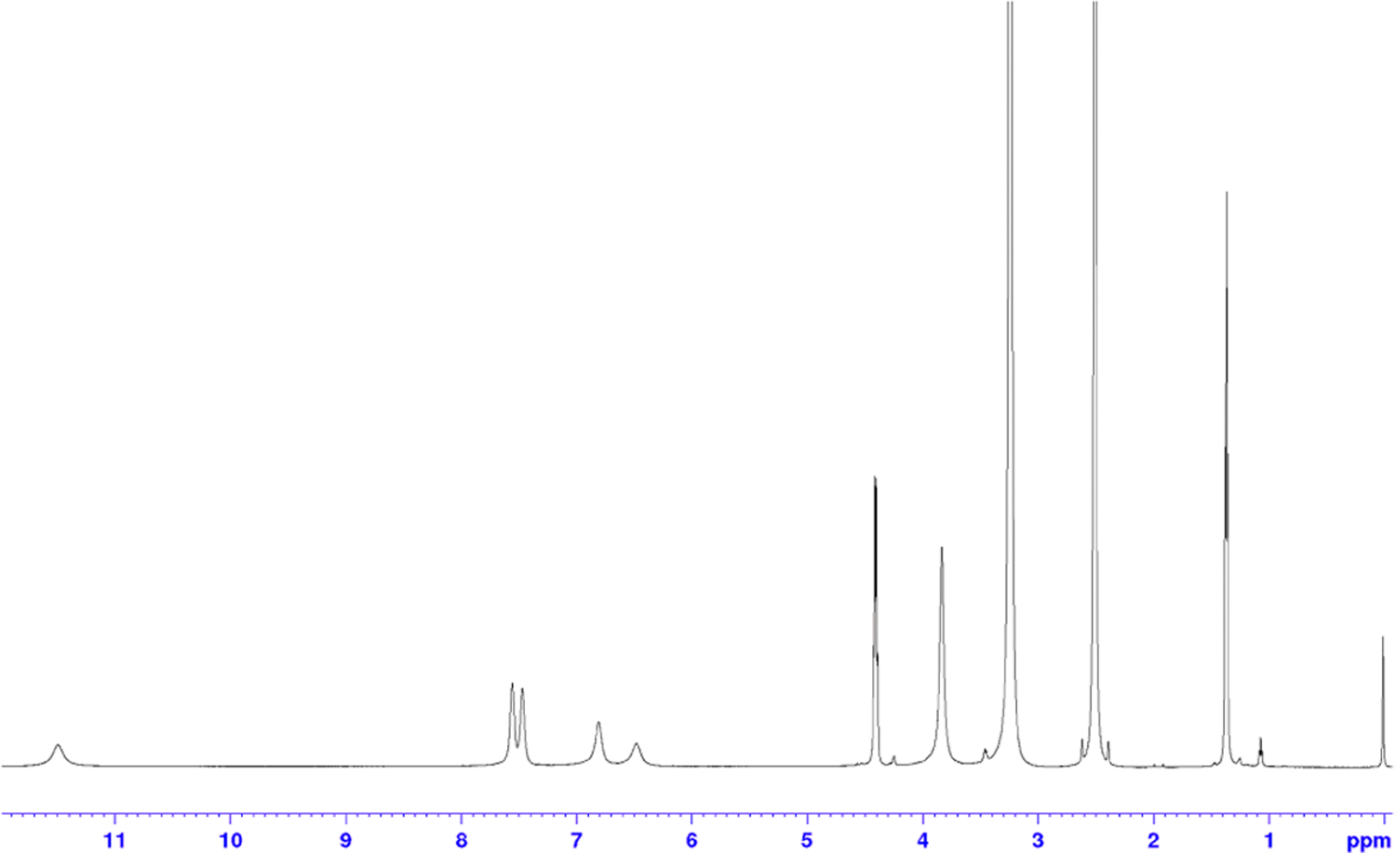
^1^H-NMR spectrum of **14a** in DMSO-*d*_6_ at 310 K, with a broad signal visible and a lack of multiplicity.

In the case of the regioisomer pair **14a–b** (Table 4), the use of green substitute solvents, namely, GVL and DEC, was possible. In these solvents, conversions were higher or equal to those in DCB (entry 1). It was also demonstrated that by changing the composition of the binary solvent, a significant change can be achieved in the regioisomeric ratios. More specifically, a regioisomeric ratio of approximately [2.38;3.32]:1 (entry 1) can be changed to approximately [1.10;1.20]:1 (entry 5), while slightly increasing the dissolved quantity of **14a**. It is important to note that in the scientific literature, the 7-substituted regioisomer **14b** was the most favored product, with the highest regioselectivity toward the 7-methoxysubstituted product.^34^ With the proposed tuneable solvent systems, a major increase was achieved in the ratio of the 5-substituted product (entry 5). Compound **14a** has not been thoroughly characterized in the literature. A major anomaly was found while analyzing its NMR spectra, measured in DMSO-*d*_6_, wherein a lack of multiplicity and the broadness of signals made the spectrum inadequate (Figure 4).

**Table 4:** Molar Percentage of Dissolved Products Compared to the Corresponding Starting Aniline Derivatives [%] in the Synthesis of Compounds 14a–b and 15a–b in DCB, GVL, DEC, V27, and V60 Solvents, Measured with Densitometry and Quantitative NMR of the Crude Products

			dissolved product [%]					
			regioisomer a		regioisomer b		regioisomeric ratio [*n*_b_/*n*_a_]	
product pairs	entry #	solvent system	qNMR	DM	qNMR	DM	qNMR	DM
14	1	DCB	11.26	9.43	26.8	31.28	2.38	3.32
	2	GVL	19.08	25.55	66.29	68.63	3.47	2.69
	3	DEC	no trace of product				n.a.^a^	n.a.^a^
	4	V27	13.09	12.88	27.19	23.8	2.08	1.85
	5	V60	25.66	23.9	28.21	28.6	1.10	1.20
15	6	DCB	21.17	20.54	19.23	19.77	0.91	0.96
	7	GVL	n.a.^b^	22.91	13.44	17.85	n.a.^c^	0.78
	8	DEC	n.a.^b^	7.05	6.46	8.71	n.a.^c^	1.23
	9	V27	n.a.^b^	10.49	15.30	12.78	n.a.^c^	1.21
	10	V60	n.a.^b^	19.28	19.6	21.12	n.a.^c^	1.09

a Regioisomeric ratio cannot be determined due to a lack of products.

b Signals of protons could not be assigned due to overlapping peaks in the ^1^H NMR spectra of crude products.

c Regioisomeric ratio cannot be determined by qNMR due to the unreliability of the spectra.

**Figure 5:**
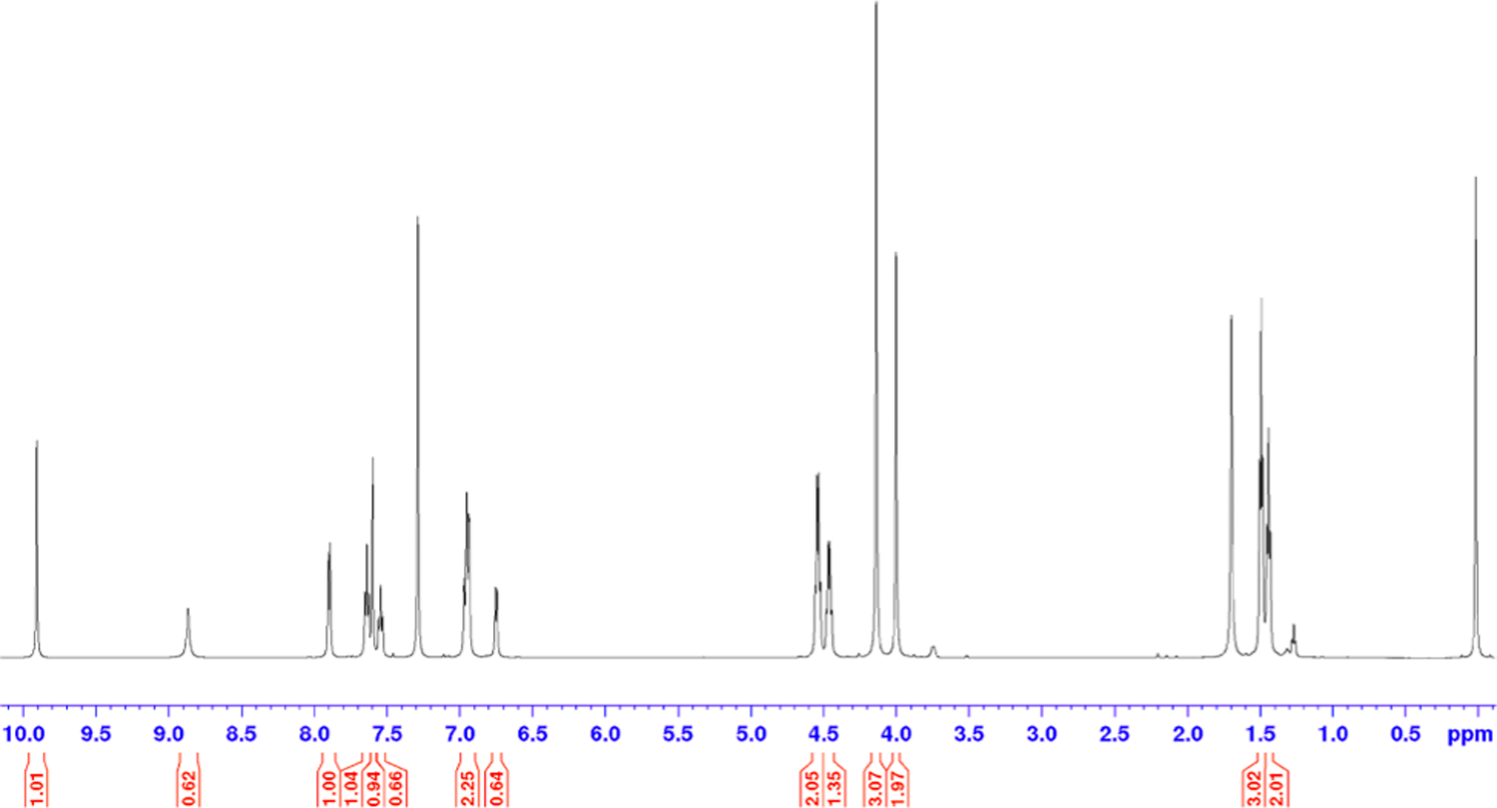
^1^H-NMR spectrum of **14a** in CDCl_3_ at 285 K, with adequate multiplicity and the two tautomers visible.

A chemical equilibrium was hypothesized between the enolic **14aA** and its oxo-tautomer **14aB**. New measurements were performed at 285 K in CDCl_3_ (Figure 5).

**Figure 6:**
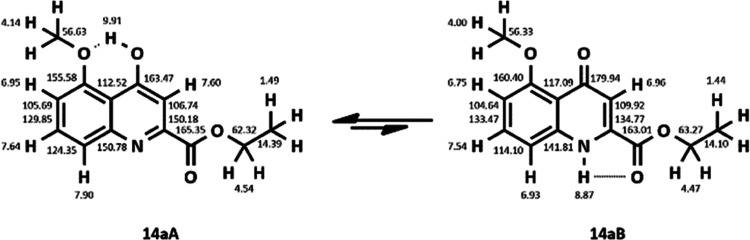
Oxo–enol tautomerism of **14a**, with full signal assignment.

The latter measurements supported the hypothesis that, at 285 K, in chloroform, a chemical equilibrium is achieved between the enolic **14aA** (the major tautomer) and the oxo-form **14aB** (the minor tautomer) with a ratio of 1:∼0.6. The structures of the two tautomers were proved by the assignment of protons and carbon atoms by ^1^H, ^13^C, and 2D (NOESY, HSQC, HMBC) NMR analyses (Figure 6).

The tautomeric ratio can be explained by a hydrogen bond formation of the hydroxylic hydrogen (δ 9.91) and the oxygen of the C5-methoxy group, forming a nonstrained six-membered ring in the **14aA** tautomer. However, in **14aB**, only a more strained five-membered ring can be hypothesized by the formation of a hydrogen bond between the N–H hydrogen (δ 8.87) and the oxo-group of the ethoxy–carbonyl function.

Analysis of the regioisomer pair **15a–b** proved that the regioisomer ratio can be modified from 0.78:1 (entry 7) to 1.23:1 (entry 8), although compound **15b** had the highest dissolved quantity in V60, with a regioisomeric ratio of 1.09 (entry 10). We presume that the difference between the regioisomeric ratios of the two compound pairs is rooted in the substituent effect. The chloro substituent is an electron-withdrawing group, with no ability to form hydrogen bonds, while the methoxy moiety is a hydrogen-bond acceptor with a high potential to form secondary bonds with the solvents either intra- or intermolecularly. Furthermore, it has an electron-donating effect on the aromatic ring. The methoxy group has a higher steric hindrance than the chloro substituent. Consequently, compound **14b** is the more favored product (entry 2). The ability to interfere with the ratio is more apparent in the case of compound **14** than in **15**, which can be explained by the ability of hydrogen-bond formation and the steric hindrance of the methoxy group.

It can be deduced that in the synthesis of kynurenic acid and its derivatives (**7–9**, **14a–b**, and **15a–b**) prepared via the CL method, GVL and DEC can serve as green substitutes for DCB (and other nonrenewable solvents) with a possibility to change the regioisomeric ratios.

### 2.3 Scale-Up and Isolation

After determining the optimal reaction conditions, major scale-up and adequate isolation were conducted, resulting in the formation of gram-scale products **7–9**, **14a–b**, and **15a–b**. Conditions of the scale-up procedures remained the same as those of the test reactions, with only the reaction volume being modified to 25 mL. Methods of isolation and yield are discussed in the Experimental Section.

## 3 Conclusions

Crucial parameters including the reaction time, reaction temperature, solubility, concentration, and molar ratio of substrates along with the overall reaction volume in the synthesis of five kynurenic acid derivatives were determined, investigated, and optimized for one-batch, two-step reactions. Four green, tuneable solvent systems made of DEC and GVL were introduced as substitutes for DCB as the reaction medium in each synthesis. The performance of the green solvent systems was highlighted because of their ability to be used in microwave-assisted reactions. Yields are not lower than those found in traditional solvents. Moreover, the potential of the tuneable solvent system to regulate regioisomeric ratios in CL synthesis was emphasized.

It should be emphasized that both the synthetic method itself and the analytical technique were designed on the basis of the concept of green chemistry. The benefits of TLC densitometry, a rapid, eco-friendly, and inexpensive analytical technique, for reaction monitoring and conversion analysis compared to quantitative NMR were revealed.

A gram-scale scale-up was accomplished in the halogenated solvent DCB and, most importantly, in green substitutes, with the solvent performance similar to that in preliminary experiments. These synthetic methods facilitated the CL synthesis of unsubstituted and several substituted kynurenic acid analogues.

## 4 Experimental Section

### 4.1 General Methods for the Test Reactions

The corresponding aniline derivative (2.5 mmol of 4–6, 10, 11) was dissolved in or mixed with the chosen solvent (DCB, GVL, DEC, V27, or V60), yielding about 3.8–3.9 mL of solutions in a 10 mL tubular MW vessel; then, 436 microliters (1.09 equiv) of DEAD (CAS: 762-21-0; purchased from Sigma-Aldrich) was added in three portions into the stirred reaction. Finally, the reaction volume was topped up with the selected solvent, yielding exactly 5.0 mL of reaction media. Reactions were conducted in a CEM Focused Microwave Synthesis System, Discover SP, with conditions discussed in the “Results and Discussion” section.

### 4.2 General Methods for Scale-Up Reactions

The aniline derivatives (12.5 mmol of **4–6**, **10**, **11**) were dissolved in, or mixed with, the corresponding solvents (procedure A: DCB; procedure B: green substitutes GVL, DEC, V27, or V60, in which the test reactions gave the highest forecasted yield), yielding about 20 mL of solutions in a 35 mL tubular MW vessel; then, 2180 μL (1.09 equiv) of DEAD (CAS: 762-21-0; purchased from Sigma-Aldrich) was added in three portions into the stirred reaction mixture. Finally, the reaction volume was topped up with the selected solvent, giving exactly 25.0 mL of reaction media. Reactions were conducted in a CEM Focused Microwave Synthesis System, Discover SP, with the reaction conditions set on the basis of test reactions (temperature 120 °C for step (i) and 180 °C for step (ii)).

Melting points were determined on a Hinotek X-4 melting point apparatus.

#### 4.2.1 Ethyl 4-Oxo-1,4-dihydroquinoline-2-carboxylate (**7**)

Procedure A: Preparation according to the general procedure, with a reaction time of 60 min for step (i), 60 min for step (ii), and 1,2-DCB as the solvent. Work-up: crystals, formed after cooling the reaction, were filtered and washed with 15 mL of diethyl ether. Yield: 1.11 g (41%).

Procedure B: Preparation according to the general procedure, with a reaction time of 60 min for step (i), 60 min for step (ii), and GVL as the solvent. Work-up: after cooling the reaction, 3 mL of DEC was added, and the product was crystallized, filtered, and washed with 15 mL ethyl acetate. Yield: 1.14 g (42%).

M.p. 209–211 °C (lit. 210–212)^32,34^

#### 4.2.2 Ethyl 6-methyl-4-oxo-1,4-dihydroquinoline-2-carboxylate (**8**)

Procedure A: Preparation according to the general procedure, with a reaction time of 90 min for step (i), 120 min for step (ii), and 1,2-DCB as the solvent. Work-up: crystals, formed after cooling the reaction, were filtered and washed with 15 mL of diethyl ether. Yield: 1.07 g (37%).

Procedure B: Preparation according to the general procedure, with a reaction time of 90 min for step (i), 120 min for step (ii), and V60 as the solvent. Work-up: after cooling the reaction, 3 mL of DEC was added, and the product was crystallized, filtered, and washed with 15 mL of ethyl acetate. Yield: 1.53 g (53%).

M.p. 211–213 °C (lit. 209–217)^32,34^

#### 4.2.3 Ethyl 6-Methoxy-4-oxo-1,4-dihydroquinoline-2-carboxylate (**9**)

Procedure A: Preparation according to the general procedure, with a reaction time of 60 min for step (i), 60 min for step (ii), and 1,2-DCB as the solvent. Work-up: crystals, formed after cooling the reaction, were filtered and washed with 15 mL of diethyl ether. Yield: 1.57 g (51%).

Procedure B: Preparation according to the general procedure, with a reaction time of 60 min for step (i), 60 min for step (ii), and V27 as the solvent. Work-up: after cooling the reaction, 3 mL of DEC was added, and the product was crystallized, filtered, and washed with 15 mL of ethyl acetate. Yield: 1.64 g (53%).

M.p. 223–224 (lit. 222–224 °C)^30,34,41^

#### 4.2.4 Ethyl 4-Hydroxy-5-methoxyquinoline-2-carboxylate (**14aA**) and Ethyl 5-Methoxy-4-oxo-1,4-dihydroquinoline-2-carboxylate (**14aB**)

Procedure A: Preparation according to the general procedure, with a reaction time of 90 min for step (i), 60 min for step (ii), and 1,2-DCB as the solvent. Work-up: regioisomer **14b** was crystallized from the reaction mixture via cooling. After filtration, the filtrate was distilled and purified by column chromatography (eluent = *n*-hexane/EtOAc 1:2), crystallized with 10 mL of diethyl ether, and washed with 5 mL of diethyl ether. Yield: 0.25 g (8%).^35^

Procedure B: Preparation according to the general procedure, with a reaction time of 90 min for step (i), 60 min for step (ii), and GVL as the solvent. Work-up: regioisomer **14b** was crystallized from the reaction mixture by the addition of 3 mL of DEC and cooling. After filtration, the filtrate was distilled and purified by column chromatography (eluent = *n*-hexane/EtOAc 1:2), crystallized with 10 mL of ethyl acetate, and washed with 10 mL of ethyl acetate. Yield: 0.68 g (22%).^35^

M.p. 215–217 °C

#### 4.2.5 NMR Spectrum of Tautomer **14aA**

^1^H NMR (CDCl_3_) δ 1.49 (3H, t, *J* = 7.2 Hz); 4.14 (3H, s); 4.54 (2H, q, *J* = 7.2 Hz); 6.95 (1H, d, *J* = 8.2 Hz); 7.60 (1H, s); 7.64 (1H, t, *J* = 8.4 Hz); 7.90 (1H, d, *J* = 8.5 Hz); 9.91 (1H, s); ^13^C NMR (CDCl_3_); 14.39; 56.63; 62.32; 105.69; 106.74; 112.52; 124.35; 129.85; 150.18; 150.78; 155.58; 163.47; 165.35.

#### 4.2.6 NMR Spectrum of Tautomer **14aB**

^1^H NMR (CDCl_3_) δ 1.44 (3H, t, *J* = 7.1 Hz); 4.00 (3H, s); 4.47 (2H, q, *J* = 7.1 Hz); 6.75 (1H, d, *J* = 8.2 Hz); 6.96 (1H, s); 7.54 (1H, t, *J* = 8.4 Hz); 6.93 (1H, d, *J* = 8.3 Hz); 8.87 (1H, s); ^13^C NMR (CDCl_3_); 14.10; 56.33; 63.27; 104.64; 109.92; 114.10; 117.09; 133.47; 134.77; 141.81; 160.40; 163.01; 179.94.

#### 4.2.7 Ethyl 7-Methoxy-4-oxo-1,4-dihydroquinoline-2-carboxylate (**14b**)

Procedure A: Preparation according to the general procedure, with a reaction time of 90 min for step (i), 60 min for step (ii), and 1,2-DCB as the solvent. Work-up: crystals, formed by cooling the reaction mixture, were filtered and washed with 15 mL of diethyl ether. After filtration, the filtrate was distilled and purified by column chromatography (eluent = *n*-hexane/EtOAc 1:2), crystallized with 10 mL of diethyl ether, and washed with 5 mL of diethyl ether. Yield: 0.87 g (28%).

Procedure B: Preparation according to the general procedure with a reaction time of 90 min for step (i), 60 min for step (ii), and GVL as the solvent. Work-up: after cooling the reaction, 3 mL of DEC was added, and the product was crystallized, filtered, and washed with 15 mL of ethyl acetate. After filtration, the filtrate was distilled and purified by column chromatography (eluent = *n*-hexane/EtOAc 1:2), crystallized with 10 mL of ethyl acetate, and washed with 5 mL of ethyl acetate. Yield: 1.91 g (62%).

M.p. 214–215 °C (lit. 215–216 °C)^34^

#### 4.2.8 Ethyl 5-Chloro-4-oxo-1,4-dihydroquinoline-2-carboxylate (**15a**)

Procedure A: Preparation according to the general procedure, with a reaction time of 120 min for step (i), 60 min for step (ii), and 1,2-DCB as the solvent. Work-up: regioisomer **15b** was crystallized from the reaction mixture by cooling. After filtration, the filtrate was distilled and purified by column chromatography (eluent = *n*-hexane/EtOAc/DCM 1.5:1:2), crystallized with 15 mL of diethyl ether, and washed with 5 mL of diethyl ether. Yield: 0.81 g (25%).

Procedure B: Preparation according to the general procedure, with a reaction time of 120 min for step (i), 60 min for step (ii), and GVL as the solvent. Work-up: regioisomer **15b** was crystallized from the reaction mixture by the addition of 3 mL of DEC and cooling. After filtration, the filtrate was distilled and purified by column chromatography (eluent = *n*-hexane/EtOAc/DCM 1.5:1:2), crystallized with 10 mL of ethyl acetate, and washed with 5 and 10 mL of ethyl acetate. Yield: 0.84 g (26%).

M.p. 200–202 °C (lit. 197–205 °C)^31,36^

#### 4.2.9 Ethyl 7-Chloro-4-oxo-1,4-dihydroquinoline-2-carboxylate (**15b**)

Procedure A: Preparation according to the general procedure, with a reaction time of 120 min for step (i), 60 min for step (ii), and 1,2-DCB as the solvent. Work-up: crystals were formed by cooling the reaction mixture, filtered, and washed with 15 mL of diethyl ether. After filtration, the filtrate was distilled and purified by column chromatography (eluent = *n*-hexane/EtOAc/DCM 1.5:1:2), crystallized with 15 mL of diethyl ether, and washed with 5 mL of diethyl ether. Yield: 0.81 g (25%).

Procedure B: Preparation according to the general procedure with a reaction time of 120 min for step (i), 60 min for step (ii), and V60 as the solvent. Work-up: after cooling the reaction, 3 mL of DEC was added, and the product was crystallized, filtered, and washed with 15 mL of ethyl acetate. After filtration, the filtrate was distilled and purified by column chromatography (eluent = *n*-hexane/EtOAc/DCM 1.5:1:2), crystallized with 10 mL of ethyl acetate, and washed with 5 and 10 mL of ethyl acetate. Yield: 0.81 g (25%).

M.p. 255–257 °C (lit. 250–259 °C)^31,36,38^

### 4.3 Quantitative NMR—Sample Extraction, Addition of Internal Standard, and Measurement

First, 250 μL of homogenous samples were extracted from the reaction media. For the reference signal and the signals of analytes to be commensurate, in the case of compounds **7–9**, 20.0 mg, and, in the case of compound pairs **14** and **15**, 10.0 mg of PAA were added to each sample.

All samples were diluted with DMSO-*d*6, giving 600 μL of solutions, measurable at room temperature (RT), on a Bruker DRX-500 spectrometer (Bruker Biospin, Karlsruhe, Baden Württemberg, Germany) at 500 MHz (^1^H), with the deuterium signal of the solvent as the lock and TMS as the internal standard (^1^H).

### 4.4 Structural Analysis Of Compound **14a**

Structural elucidation was conducted *via*^1^H, ^13^C, and 2D (NOESY, HSQC, HMBC) NMR analyses at 285 K in CDCl_3_ as the solvent on a Bruker AVANCE III 600 MHz spectrometer at 600 (^1^H) and 150 (^13^C) MHz, with the deuterium signal of the solvent as the lock and TMS as the internal standard (^1^H, ^13^C) (Figures S2–S6).

### 4.5 TLC Densitometric Analysis

#### 4.5.1 Sample Extraction

From each reaction medium, 0.5 mL was extracted and diluted with ethanol in two to three steps (in the case of compound pair **15**, with three drops of DMSO additive). Using ethanol is beneficial due to its natural occurrence, good ESH profile, moderate volatility, and inability of transesterification.

#### 4.5.2 Calibration

Dilution series were prepared of pure stocks of each product compound. The calibration range was precisely set by virtue of the linearity range of the γ-curve. 5 to 10 spots (depending on the linearity range) were applied to the plate.

Individual ranges of measurements were assigned and are presented in Table 5.

#### 4.5.3 Application and Elution

On each TLC plate, 2 μL (in the case of DEC media, due to a low predicted yield, 4 or 6 μL) of solutions (5–10 of reference and 5 of analyte) were applied, resulting in 10 to 15 spots, with about 0.3 cm diameter, 1.0 cm from the bottom of the plate, and 1.0 cm between each other. After evaporating the solvent, the plates were developed in adequate eluents (Table 5) (50 mL of eluent in a glass elution chamber of 20/20/10 cm [*w*/*h*/*d*] dimensions) and then left to dry for half an hour at room temperature.

**Table 5:** Ranges of Measurements and Eluents of the TLC Analysis of the Corresponding KYNA Analogues

	range of measurement		
compound	minimum (ng/spot)	maximum (ng/spot)	eluent
7	3.3	33	*n*-hexane:EtOAc
8	45.0	450	1:1
9	20.0	200	
14a	1.34	13.4	*n*-hexane:EtOAc
14b	8.56	42.8	1:2
15a	9.88	98.8	*n*-hexane:EtOAc:DCM
15b	20.92	104.6	1.5:1:2

#### 4.5.4 Recording the Plates

The plates were placed into a thoroughly obscured plastic black chamber (60/20/40 cm [*w*/*h*/*d*]) with a light-absorbing bottom. While recording the TLC plates, UV derivatization at 366 nm was applied as the Zn_2_SiO_4_ (F254) content of the SiO_2_ plate has low light emission or absorbance at this wavelength. Moreover, the products have a high emission-to-absorbance ratio (compared to 254 nm). For recording the plates, an everyday smartphone, the Xiaomi Redmi Note 8T (48-megapixel mode, manual focus, ISO 100, 1/200 to 16 s exposure) and a Fujifilm FinePix S5800 digital camera (8-megapixel mode, manual focus, ISO 100, 1/200 to 4 s exposure) were used. Records were quantified with ImageJ software on a blue channel, giving the best signal-to-noise ratio.

## Supporting Information Available

^1^H-, ^13^C-, and 2D (HSQC, HMBC, NOESY) NMR characterizations of compound **14a** (with tautomers **14aA** and **14aB**) (PDF)

## Abbreviations

*a*MA: *aza*-Michael addition
CL: Conrad–Limpach
DCB: 1,2-dichlorobenzene
DEAD: diethyl-acetylenedicarboxylate
DEC: diethyl-carbonate
DM: densitometry
GVL: γ-valerolactone
qNMR: quantitative nuclear magnetic resonance spectroscopy
TLC: thin-layer chromatography
TRC: thermal ring closure
