# Supplementary material for: Novel Eco-friendly, One-Pot Method for the Synthesis of Kynurenic Acid Ethyl Esters
Source: ACS Omega. 2023 May 11;8(20):17966–75. doi: 10.1021/acsomega.3c01170 (PMC10210203; doi:10.1021/acsomega.3c01170)
Supplement: Supplementary file 1 — ao3c01170_si_001.pdf [file ao3c01170_si_001.pdf]

A novel eco-friendly, one-pot method for the synthesis of kynurenic acid ethyl esters

Péter Simon[a], Bálint Lőrinczi[a], Anasztázia Hetényi[b] and István Szatmári[a,c,*]

[a] Institute of Pharmaceutical Chemistry, University of Szeged, Eötvös u. 6, H-6720 Szeged, Hungary

[b] Department of Medical Chemistry, University of Szeged, Dóm tér 8, H-6720 Szeged, Hungary

[c] Stereochemistry Research Group, Eötvös Loránd Research Network, University of Szeged, Eötvös u. 6, H-6720 Szeged, Hungary

* Correspondence: szatmari.istvan@szte.hu

Contents

1. ^{1}H NMR (DMSO-*d6* and $CDCl_3$) spectra of compound **14a** S2

2. ^{13}C NMR spectrum of compound **14a** S3

3. NOESY spectrum of compound **14a** S3

4. HSQC spectrum of compound **14a** S4

5. HMBC spectrum of compound **14a** S4

Ethyl 4-hydroxy-5-methoxyquinoline-2-carboxylate (**14aA**) and *Ethyl 5-methoxy-4-oxo-1,4-dihydroquinoline-2-carboxylate* (**14aB**)

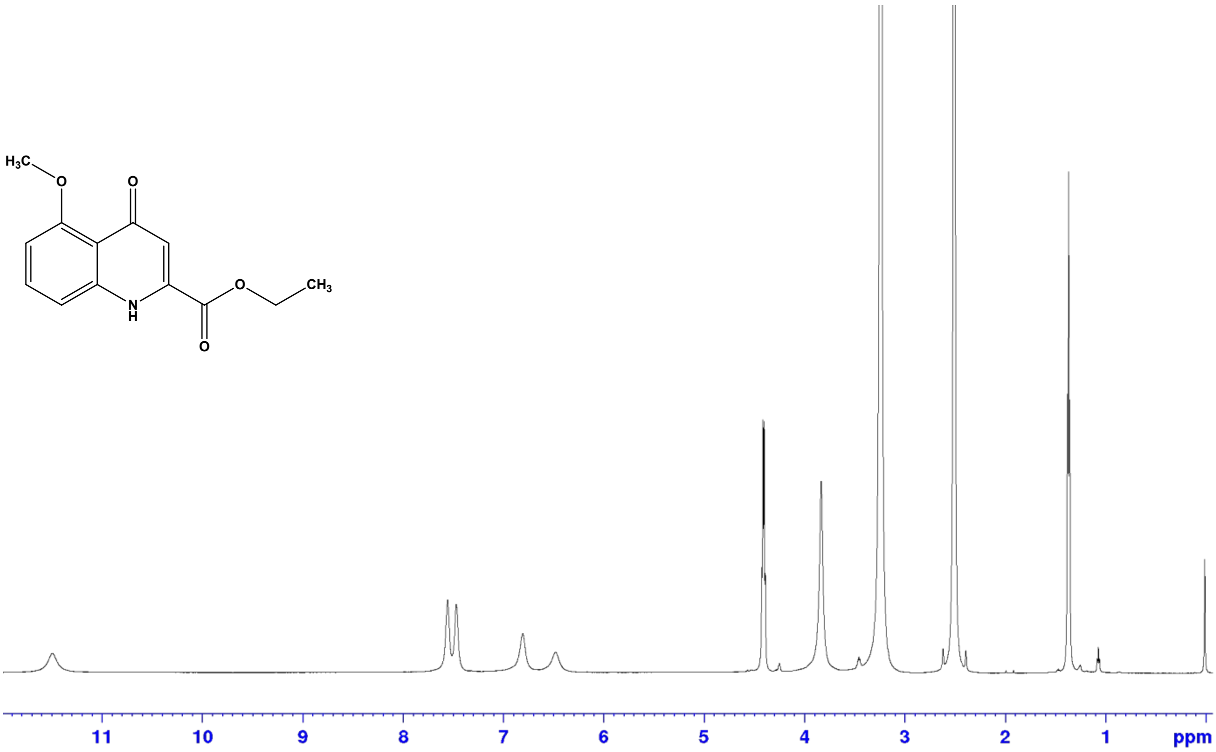

Figure S1. ^{1}H NMR spectrum of **14a** in DMSO-*d6,* at 310 K

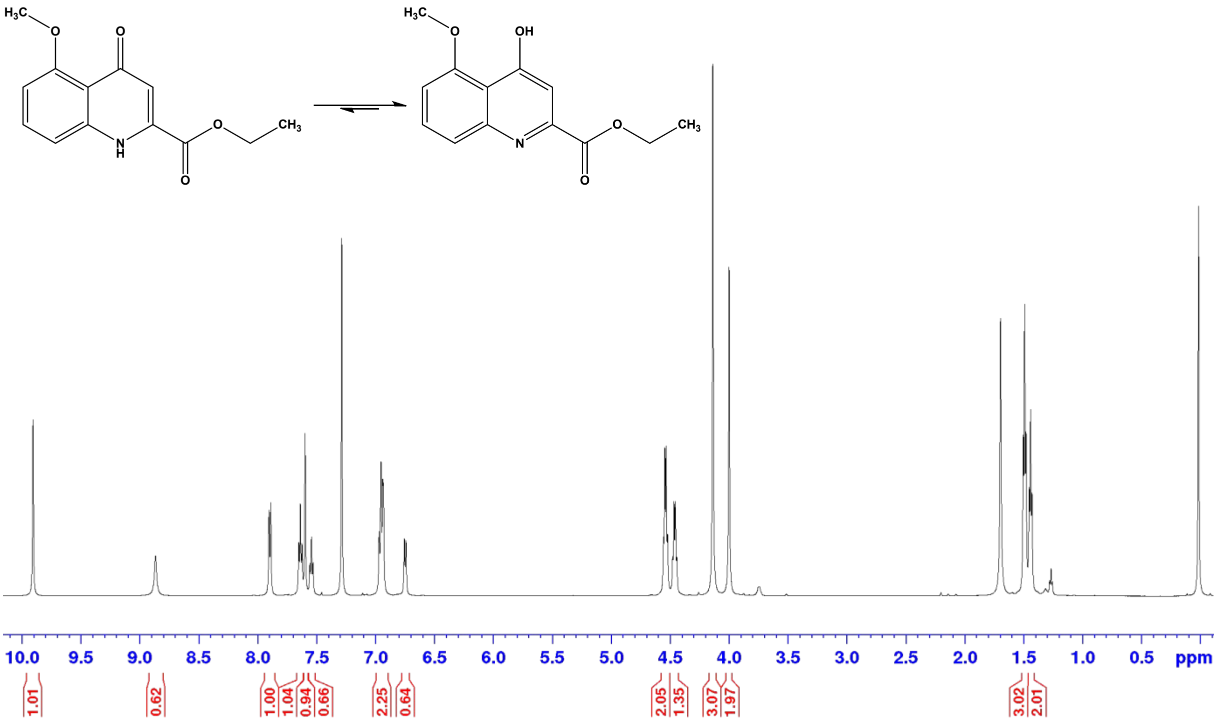

Figure S2. ^{1}H NMR spectrum of **14a** in $CDCl_3$, at 285 K – the two tautomers **14aA** and **14aB** are visible

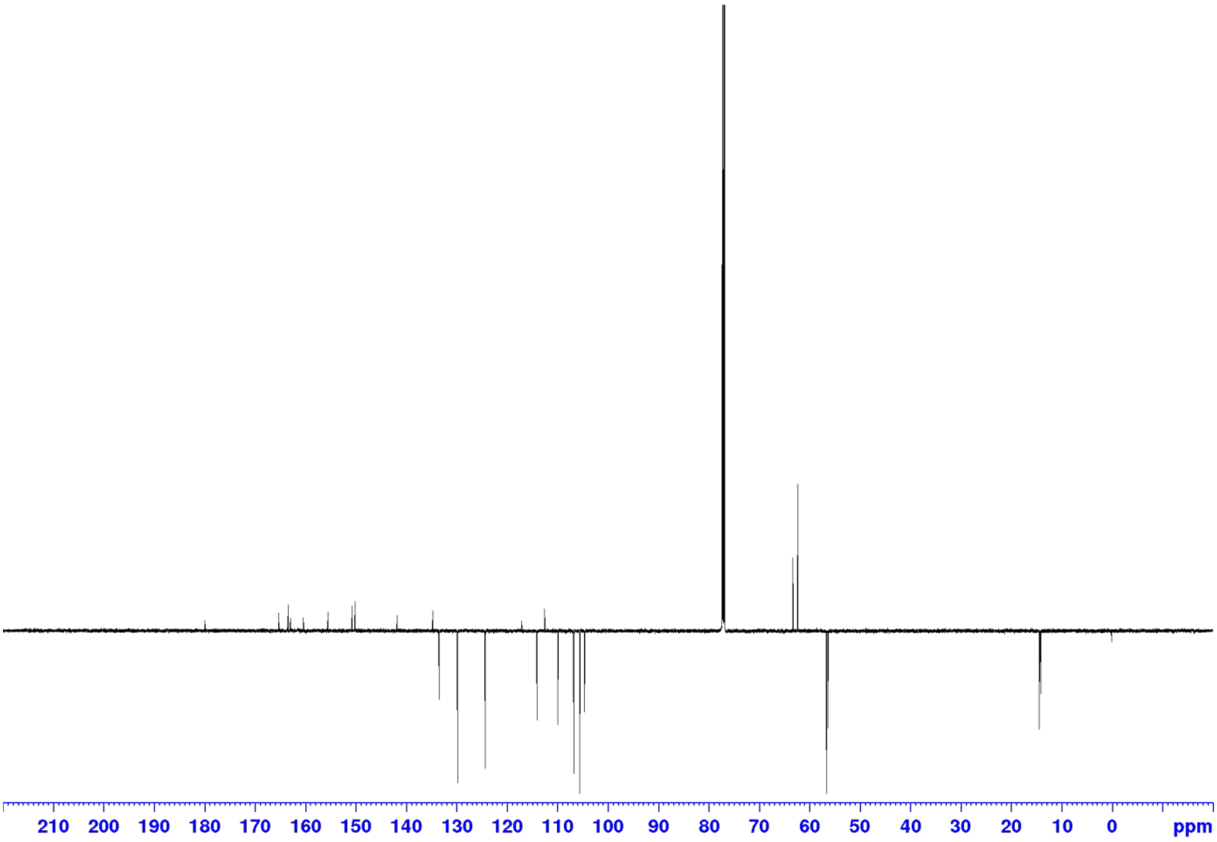

Figure S3. ^{13}C NMR spectrum of **14a** in $CDCl_3$, at 285 K – the two tautomers **14aA** and **14aB** are visible

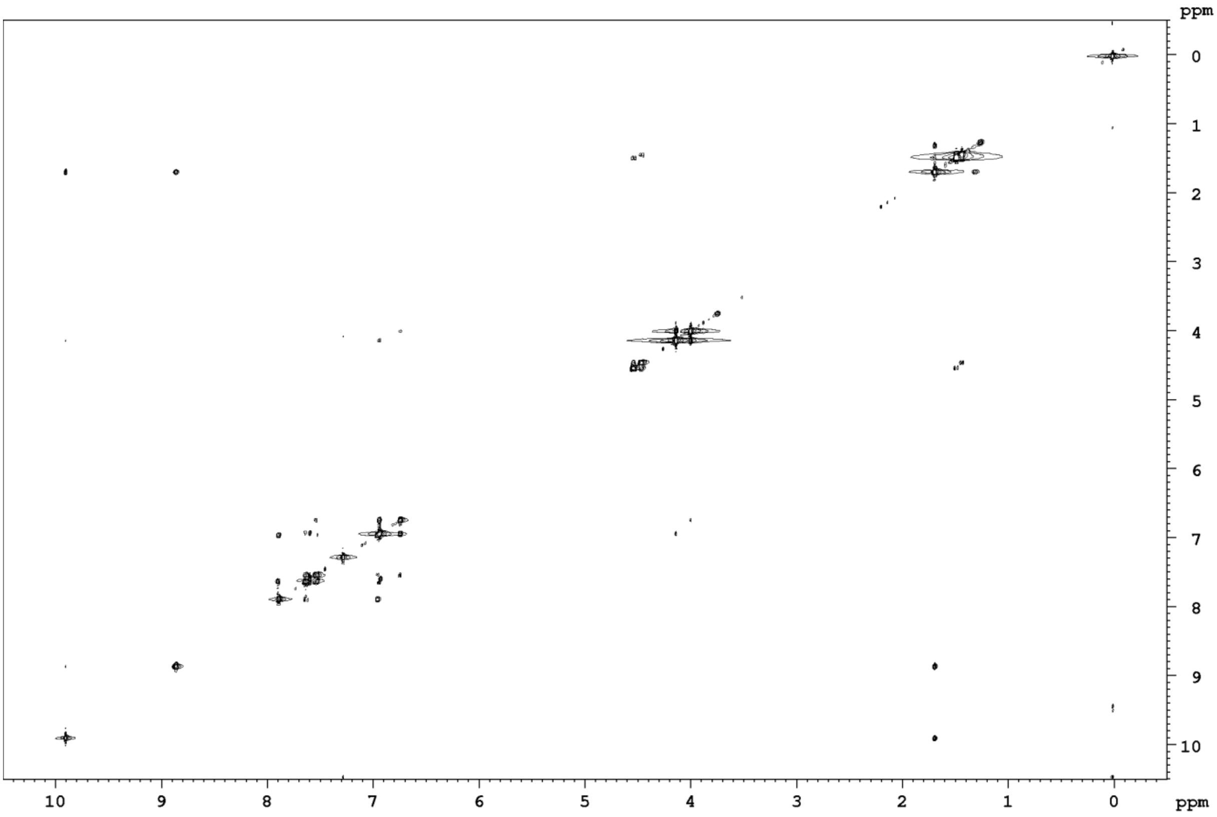

Figure S4. NOESY spectrum of **14a** in $CDCl_3$, at 285 K – the two tautomers **14aA** and **14aB** are visible

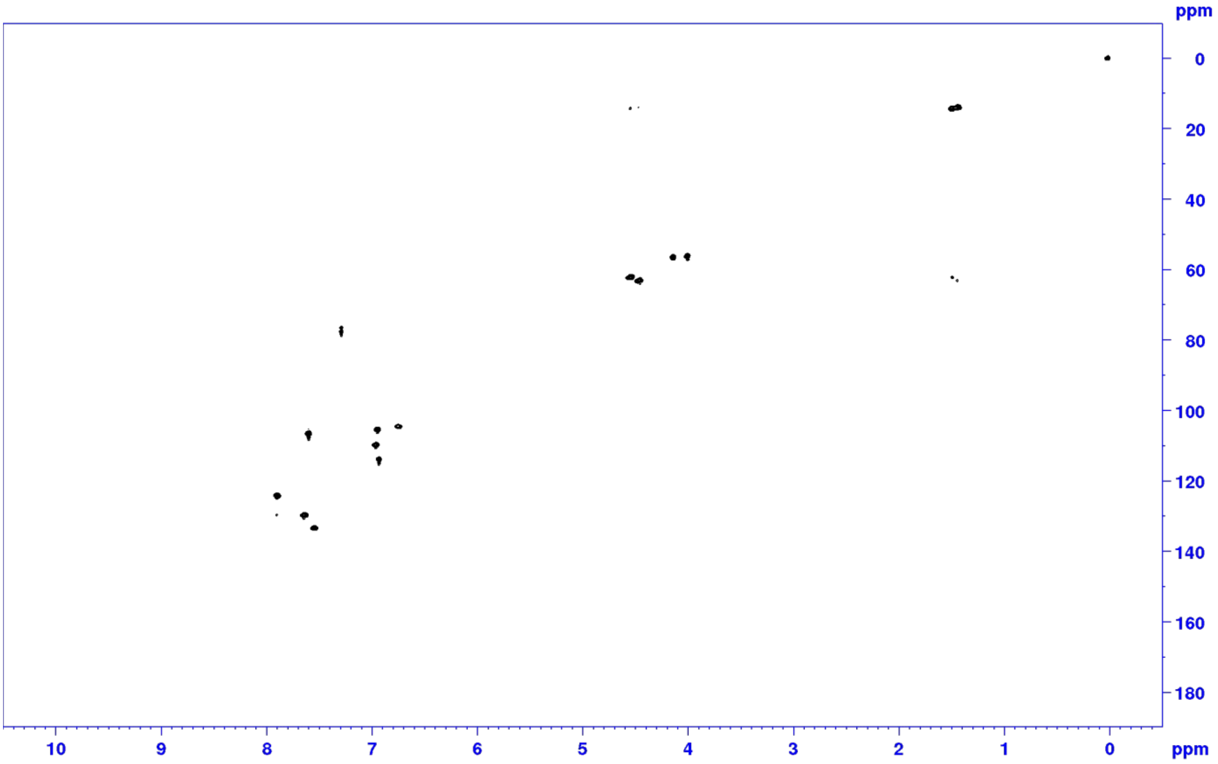

Figure S5. HSQC spectrum of **14a** in $CDCl_3$, at 285 K – the two tautomers **14aA** and **14aB** are visible

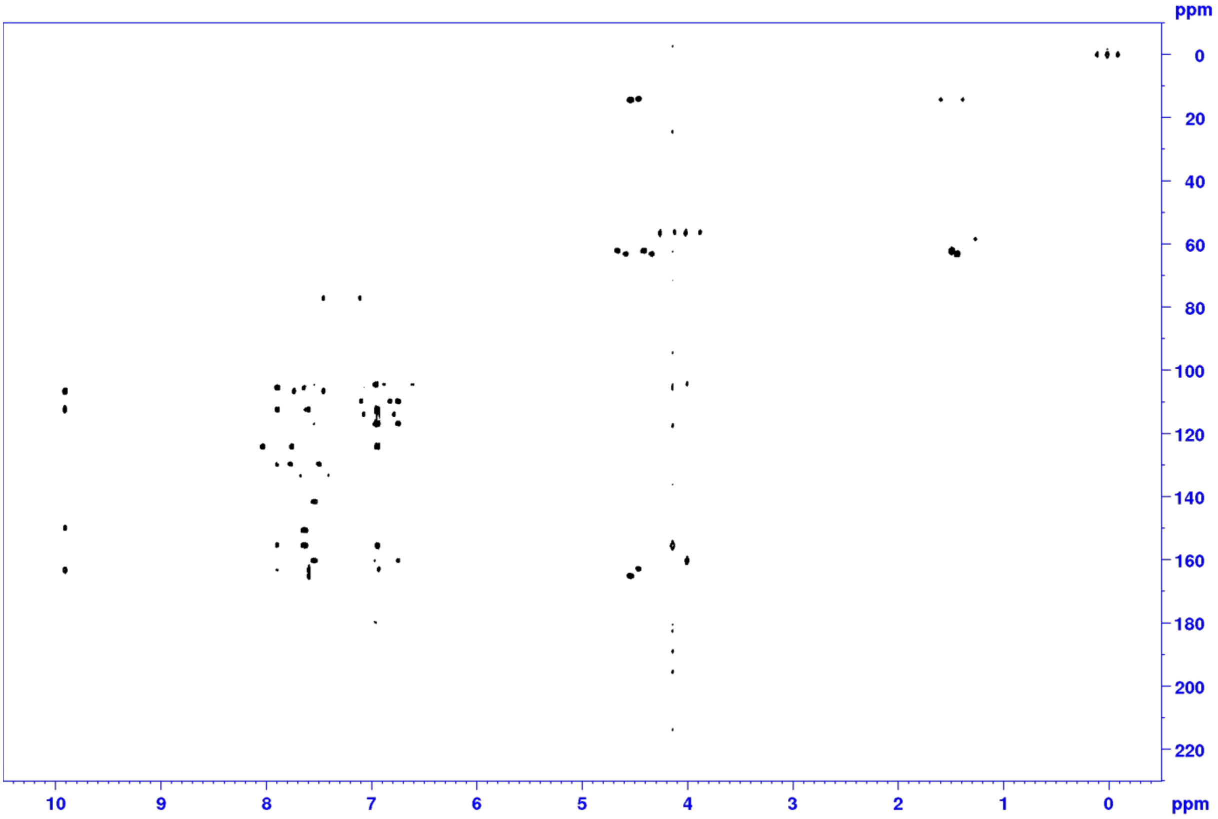

Figure S6. HMBC spectrum of **14a** in $CDCl_3$, at 285 K – the two tautomers **14aA** and **14aB** are visible
